# Medical Residency – where we are and future challenges

**DOI:** 10.1590/1516-3180.2025.1434.14042025

**Published:** 2025-07-14

**Authors:** Alfredo Inácio Fiorelli, Paulo Manuel Pêgo-Fernandes

**Affiliations:** I Professor, General Coordinator, Medical Residency Commission (COREME), Faculty of Medicine, Universidade de São Paulo (USP), São Paulo (SP), Brazil.; II Vice Dean, Faculdade de Medicina, Universidade de São Paulo (USP), São Paulo (SP), Brazil; Department of Cardiopneumology, Faculdade de Medicina, Universidade de São Paulo (USP), São Paulo (SP), Brazil; Full Professor; Director of the Scientific Department, Associação Paulista de Medicina (APM), São Paulo (SP), Brazil.

 The concept of medical residency has its roots in ancient medicinal practices, such as in Greece, where teachings are transmitted by experienced professionals through practical observation. This informal system persisted until the emergence of universities in the Middle Ages, when practical and theoretical training was introduced.^1,2^ Notably, the University of Bologna in 1088 and the University of Paris in 1150. 

 In the mid-19th century, Johns Hopkins Hospital became the cornerstone medical residency program. Notable pioneers in this program, such as William Halsted (1852–1922) and William Osler (1849–1919), emphasized a humanistic approach with direct patient observation and mentorship. In this innovative teaching model, residents began living in the hospital, hence the term "residency." The model evolved with emphasis on competency-based education, interdisciplinary collaboration, and technological advances.^3^

 Medical residency represents a fundamental stage of a physician’s education, consolidating the theoretical learning acquired in medical schools and promoting the development of practical, ethical, and human competencies essential to medical practice. It is an intensive educational process based on the principle of in-service education, in which the resident participates in real-world clinical and hospital environments, with qualified supervision. Confronted with real challenges in healthcare, this experience not only provides technical depth in the specialties, but also sharpens clinical reasoning, responsible decision-making, and appreciation of teamwork. 

 Residents are an essential part of many healthcare teams. In teaching hospitals, they participate in strategic roles, especially in areas of high demand, human resource shortages, and regions of greater social vulnerability. Therefore, investing in well-structured residency programs is an important health indicator. 

## Initial Challenges for the Dissemination of Medical Residency

 In Brazil, medical residency was first institutionalized at the Hospital das Clínicas of the Faculty of Medicine of the Universidade de São Paulo in 1944 under the leadership of Professor Alfredo Balabram. The initial medical residency provided structured practical postgraduate training programs for newly graduated doctors. This initiative expanded into other institutions, such as the Hospital dos Servidores do Estado of Rio de Janeiro in 1948.^4^

 However, the official recognition of medical residency in Brazil occurred only in 1977, with the creation of the National Medical Residency Commission (CNRM), through Decree nº 80.281.^4^ Since then, medical residency has been the formal training pathway for medical specialization, governed by technical and ethical criteria initially established by the Ministry of Education and later by the Ministry of Health.^5^

 This model, although strongly influenced by the North American experience, has progressively incorporated the needs of the Unified Health System (SUS – *Sistema Único de Saúde*), prioritizing the training of professionals committed to our social reality. Medical residency is a consolidated model of specialized training. However, its implementation faced several obstacles, locally and internationally. The first challenge was the perceived need for medical training beyond graduation, which required cultural paradigm shifts, and institutional and legal changes. In the United States, the adoption of this teaching model was initially seen as an unnecessary extension of professional training. The consolidation of residency only gained strength with the growing need for specialist doctors after the war and with the creation of the Accreditation Council for Graduate Medical Education (ACGME) in 1981, which brought in quality assurance criteria and supervision requirements. This body is currently responsible for accrediting all medical training programs in the United States. 

 In Europe, the scenario was more heterogeneous as each country developed a different model according to its own healthcare system. A common challenge is the integration of residency into already-established hospital systems, which were often geared toward theoretical education. In the United Kingdom, the implementation of the residency model encountered substantial barriers due to the rigidity of the university system and the need to reconcile practical training with the National Health Service. Even after the creation of the European Union, diversity in medical residency models persisted. 

 The challenges in Brazil were no different. Among the initial obstacles were the scarcity of hospitals with adequate training capacity, the lack of qualified supervisors, and the need to align with the SUS. The advancement of medical residency required recognition of the complexity of modern medicine, which necessitated specialized training and supervised environments. The challenges pointed to a constant need for articulation between public policies, training institutions, and professionals committed to quality healthcare. 

 Currently, in Brazil, contrasting challenges are arising with the uncontrolled growth of medical schools, mostly private schools that lack the capacity to create and maintain their own teaching hospitals. Public hospitals often welcome teaching practices as they represent a way to sustain organizational structures and expand healthcare services. Private institutions, on the other hand, often aim to avoid the necessary infrastructural investment, which may initially seem advantageous. However, this lack soon revealed the fragility of these institutions due to a lack of long-term commitment. It is common to find students from different institutions training within the same hospital, compromising the creation of an organized teaching system. 

 It is essential that the state exercises a more effective regulatory role through the Ministry of Education and the National Medical Residency Commission. The accreditation of new programs should be conditioned on the existence of their own teaching infrastructures, with hospitals or health centers properly equipped, a minimum number of preceptors, and articulation with the local health network. Finally, it is essential that public universities, university hospitals, and medical entities take a clear and unified stance on this. Medical education is a complex process that requires time, structural resources, and institutional commitment to teaching, research, and care. 

## Adoption of Affirmative Actions

 The adoption of affirmative action based on racial and socioeconomic quotas in higher education aims to correct historical inequalities in access to professional training. Discussions on the topic are controversial and require multifaceted political action. The term ‘affirmative action’ emerged in the United States in the 1960s, aiming to reduce disparities between Whites and Blacks through government policies. The "National Resident Matching Program" encourages institutions to adopt holistic criteria for evaluating candidates, going beyond grades and scientific publications. However, in 2023, the U.S. Supreme Court banned the use of racial criteria for university admissions in favor of meritocracy, although many institutions remained committed to diversity as a strategy to reduce health disparities.^6^

 In Europe, the scenario is more conservative, and most countries prioritize academic and meritocratic criteria. However, countries such as the United Kingdom have invested in inclusion and support programs for students from vulnerable backgrounds, focusing on the equity of opportunities rather than quota reservations. The discussion of racial quotas still faces resistance, partly because of different historical and demographic contexts. 

 In Brazil, the application of racial and social quotas for undergraduate admissions has advanced, with different public institutions adopting this policy to reduce social disparities. The introduction of this policy in medical residency is much more complex and has sparked a greater debate. Opponents point out that this strategy has already been implemented at the undergraduate level, and duplication is not justified. Some public institutions have begun implementing policies to reserve a percentage of residency places for Black, Brown, and Indigenous individuals and those from public schools. The State sees these policies as positively impacting program diversity. However, the challengesincluding peer acceptance and the isolated adoption of quotas have not shown the expected effects, making the implementation of robust public policies from early educational stages necessary. 

## Challenges in the Selection Process for Medical Residents

 In the United States, the "National Resident Matching Program" is a highly competitive selection process, with a demand greater than the number of available positions. In Europe, processes vary between countries, and the lack of standardization is a challenge for professional mobility among EU member states. 

 In Brazil, the selection process is decentralized, with health institutions and universities applying their own theoretical and practical examinations, leading to multiple applications and travel demands on the candidates. Major institutions are highly competitive because of the disproportion between the available places and demand. Among other factors, the demand for positions in large urban centers discourages the placement of doctors in underserved areas, requiring additional measures. The growing demand for medical specialists requires that selection processes be not only efficient, but also fair and aligned with health system needs. 

 The assessment process must examine not only technical knowledge but also ethical decision-making and emotional behavior in high-complexity environments. However, one of the great challenges is identifying whether the resident has the right profile for the specialty, including resilience and empathy with patients, which are traits that are not always measurable through objective evaluations. During residency, programs should combine continuous and multimodal assessment methods, including self-assessment, peer evaluation, and structured direct observations. Strengthening the feedback culture and training preceptors for formative assessments are also essential strategies for addressing these challenges and ensuring excellent medical training. 

 Among various assessment tools, the Mini-Clinical Evaluation Exercise (Mini-CEX) stands out because it allows for structured evaluations of clinical performance in real time, followed by immediate feedback. This instrument is effective for assessing different aspects by observation, including patient interaction, communication skills, physical examination skills, and diagnostic formulations. 

## Future Challenges of Medical Residency

 According to the 2023 Medical Demography Report in Brazil, 4,951 accredited residency programs are offered by 789 institutions accredited by the Ministry of Education, covering 55 medical specialties and 59 areas of practice recognized by the Joint Specialties Commission.^7^

 In recent years, the number of medical residency programs has increased in an attempt to keep pace with the unregulated expansion of medical schools. Of note, approximately 60% of these programs are funded by federal, state, and municipal governments. 

 One of the main federal government initiatives is the National Program to Support the Training of Specialist Doctors in Strategic Areas (Pro-Residency), which supports the training of specialists in areas recognized as priority regions by the Unified Health System. 

 In the coming years, great challenges await. Considering that medical residency represents the most decisive stage in the training of specialist doctors. The expansion of training centers, appreciation of preceptorship, and alignment with the real needs of the Unified Health System represent important determinants for guiding future goals. 

 Another relevant challenge is the incorporation of new competencies required in today’s context. For example, the management of chronic diseases, palliative care, mental health, technology in medicine, and effective communication with patients and teams. These skills remain underexplored in medical education research. 

 Given these challenges, it is necessary to reaffirm the commitment to medical residency that trains specialists who are technically competent, ethically committed, and whose responsibilities align with real-world needs. The quality of today’s medical education defines the quality of tomorrow’s healthcare. 
